# Expectation Identities for Dynamical Systems: A Classical Analog of the Ehrenfest Theorem

**DOI:** 10.3390/e28060654

**Published:** 2026-06-09

**Authors:** Abiam Tamburrini, Sergio Davis, Diego González, Pablo S. Moya

**Affiliations:** 1 Dipartimento di Fisica, Universitá Della Calabria, I-87036 Rende, Italy; 2 Departamento de Física, Facultad de Ciencias, Universidad de Chile, Santiago 8370459, Chile; pablo.moya@uchile.cl; 3 Departamento de Física y Astronomía, Facultad de Ciencias Exactas, Universidad Andres Bello, Sazié 2212, Piso 7, Santiago 8370136, Chile; sergio.davis@cchen.cl; 4 Research Center in the Intersection of Plasma Physics, Matter and Complexity (P^2^mc), Comisión Chilena de Energía Nuclear, Casilla 188-D, Santiago 8340701, Chile; 5 Departamento de Física, Facultad de Ciencias, Universidad Católica del Norte, Av. Angamos 0610, Antofagasta 1270398, Chile; diego.gonzalez@ucn.cl

**Keywords:** non-equilibrium statistical mechanics, fluctuation–dissipation theorem, conjugate variables theorem, Liouville dynamics, Fokker–Planck dynamics, expectation value dynamics

## Abstract

In this work, we formulate a systematic expectation-value framework for dynamical systems whose probability densities evolve according to linear partial differential equations, such as the Fokker-Planck and Liouville equations. The approach is based on expectation-calculus identities associated with the Fluctuation-Dissipation Theorem and the Conjugate Variables Theorem, allowing the derivation of evolution equations directly for arbitrary observables and fluctuations without explicitly solving the full probability-density equation. The resulting relations provide a classical Ehrenfest-type formulation for observable dynamics and fluctuations under linear probability-density evolution. While the resulting equations are not closed in general, since they typically involve higher-order moments, correlations, or derivatives, the formalism offers a unified operational framework for studying observable dynamics under suitable approximations or closure assumptions. We illustrate the procedure with examples involving Fokker–Planck and Liouville dynamics and discuss the scope, limitations, and possible applications of the framework in nonequilibrium statistical mechanics. In particular, we emphasize that the method is intended as a systematic observable-based formulation for systems governed by linear evolution equations, rather than as a universal closure scheme for arbitrary nonequilibrium dynamics.

## 1. Introduction

The main language in which non-equilibrium statistical mechanics (NESM) is formulated consists of systems of partial differential equations (PDEs). Among the most important PDE systems in the field are the Fokker-Planck equation [1] and the Liouville equation [2,3], the latter including the Vlasov equation [4] as a particular case. These equations provide the time evolution of a probability density

(1) $$\rho \left(x;t\right):=P\left(X_{t}=x|I\right),$$

where $x=(x_{1},\ldots ,x_{n})\in V$ represents the degrees of freedom that characterize the non-equilibrium system under study. While the notation $P(X_{t}=x|I)$ may suggest a probability mass, we emphasize that this is a shorthand for the time-sliced probability density. As shown in González et al. [5], this density can be rigorously defined as the expectation of a Dirac delta functional over the distribution of paths,

$$\rho \left(x;t\right):=\langle \delta \left(X_{t}-x\right)\rangle$$

which corresponds to their time-slicing equation. This representation is fully consistent with the delta-projection approach used in stochastic dynamics [6]. In this sense, Equation (1) should be read as the probability density of $X_{t}$ evaluated at x, given the state of knowledge *I*, so that integration over a finite region Δx yields the probability of finding the system within Δx at time *t*. This clarification avoids the confusion between probability mass and probability density in the continuous case, while keeping the compact Bayesian notation introduced in Equation (1). Knowledge of the probability density ρ allows us to obtain different time-dependent, macroscopic quantities W(t) as expectation values of their corresponding microscopic observables w(x;t). These expectation values are given by

(2) $$W\left(t\right):={\langle w\rangle }_{t}=\int_{V}dx\;\rho \left(x;t\right)w\left(x;t\right).$$

The usual approach based on first determining the probability density and then computing all the required expectations is clear in principle, but in most cases turns out to be impractical, as we do not always have access to methods for solving the PDE for ρ in the first place.

In this work, we formulate a systematic expectation-value framework for systems whose probability densities evolve according to linear evolution equations of the form

(3) $$\partial_{t}\rho =\hat{L}\rho ,$$

including, for example, Liouville-, Fokker–Planck-, and continuity-type dynamics. Rather than focusing exclusively on standard polynomial moment hierarchies commonly used in kinetic theory and Fokker–Planck dynamics [1,7], the present framework is formulated directly in terms of arbitrary differentiable observables w(x,t) and their fluctuations.

The procedure is based on the use of two expectation identities, namely the Fluctuation–Dissipation Theorem and the Conjugate Variables Theorem [8], which provide a systematic way to relate observable dynamics to the underlying probability-density evolution. This allows one to derive evolution equations directly for physically relevant observables—including non-polynomial observables and fluctuations—without explicitly solving the full probability-density equation or constructing the hierarchy moment by moment.

While the resulting equations are not closed in general, since they typically depend on higher-order moments, correlations, or derivatives, they provide a systematic framework for studying the dynamics of specific observables. In cases where suitable assumptions, closures, or approximations are available, the method can be used to study physically relevant quantities without requiring the explicit solution of the full probability-density equation. Accordingly, the present work is not intended as a universal closure scheme, but rather as a systematic observable-based formulation from which problem-dependent approximations, reduced descriptions, or closure strategies may be constructed.

After introducing the Ehrenfest-type identity underlying the framework, which resembles the quantum-mechanical Ehrenfest theorem in a classical statistical setting, we illustrate the procedure by deriving the differential equations associated with observables under Fokker–Planck and Liouville dynamics and discuss the scope, limitations, and possible applications of the formalism in nonequilibrium statistical mechanics.

## 2. The Ehrenfest Identity for Classical Time Evolution

Consider a system with time-dependent probability distribution $\rho \left(x;t\right):=P\left(X_{t}|I\right)$, for which the time evolution equation is given by

(4) $$\partial_{t}\rho =\hat{L}\rho ,$$

where $\hat{L}$ is a linear, differential operator on the variables x, and $\partial_{t}$ represents partial derivative with respect to *t*. In the following, we assume sufficient smoothness and boundary conditions such that the integrations by parts involved in the derivations are well defined and the corresponding adjoint operator exists. Note that this is the case for all systems that follow the continuity equation [9],

(5) $$\partial_{t}\rho =-\nabla \cdot J,$$

where J(x;t)=ρv(x;t) and v(x;t) is the velocity field that describes the flow of probability. Our starting point is the general linear evolution equation for the probability density, Equation (4), which includes, as particular cases, the continuity, Liouville, and Fokker–Planck equations. It is important to mention that the continuity equation is mentioned only to illustrate that these forms share the same conservation structure, not as the basis of the derivation. In the following, we derive the following Ehrenfest-type expectation relation

(6) $$\partial_{t}{\langle w\rangle }_{t}={\langle \partial_{t}w\rangle }_{t}+{\langle {\left({\hat{L}}^{†}w\right)}^{*}\rangle }_{t},$$

valid for arbitrary differentiable observables within the class of systems governed by Equation (4), w(x;t) and where ${\hat{L}}^{†}$ is the adjoint operator to $\hat{L}$. We will refer to this as the Ehrenfest identity associated with the operator $\hat{L}$. We emphasize that Equation (6) is a formal identity; in practical applications, it typically involves expectations of derivatives of *w*, which may require further modeling or approximation to be evaluated.

The proof proceeds as follows. First, we divide both sides of Equation (4) by ρ to obtain the logarithmic form of the PDE,

(7) $$\partial_{t}\ln\rho =\frac{\hat{L}\rho }{\rho }.$$

Then, we multiply both sides by w(x;t) and take expectation under ρ, obtaining

(8) $${\langle w\;\partial_{t}\ln\rho \rangle }_{t}={\langle w\left(\frac{\hat{L}\;\rho }{\rho }\right)\rangle }_{t}.$$

where we understand that the expectation value, or simply the expectation, of any observable A(X) in the state of knowledge *I*, as we showed in Equation (2), will be written as indicated in Equation (1),

$${\langle A\rangle }_{I}=\int_{V}dxP\left(X=x|I\right)A\left(x\right).$$

Now we introduce the Fluctuation-Dissipation Theorem (FDT) for a distribution P(x|λ) as

(9) $$\partial_{\lambda }{\langle A\rangle }_{\lambda }={\langle \partial_{\lambda }A\rangle }_{\lambda }+{\langle A\partial_{\lambda }\ln P\left(x|\lambda \right)\rangle }_{\lambda }.$$

The Equation (9) is the most general form of a family of ’fluctuation theorems’. The special case where the probability distribution P(x|λ) is a Maximum Entropy model was previously derived by Jaynes in his book [10]. It is possible to recover the known fluctuation-dissipation Theorem associated with the linear response of the system, as shown in Appendix A. In the present context, the FDT provides a systematic relation between time derivatives of expectation values and expectations involving derivatives of the probability density, allowing the explicit dependence on $\partial_{t}\ln\rho$ to be rewritten in expectation-value form.

We use the Equation (9) to eliminate $\partial_{t}\ln\rho$ from the left-hand side of Equation (8), obtaining

(10) $$\partial_{t}{\langle w\rangle }_{t}-{\langle \partial_{t}w\rangle }_{t}={\langle w\left(\frac{\hat{L}\;\rho }{\rho }\right)\rangle }_{t}.$$

The remaining step consists in rewriting the right-hand side in terms of expectation values of observables by using the conjugate variables theorem (CVT), namely

(11) $${\langle {\left({\hat{L}}^{†}w\right)}^{*}\rangle }_{I}={\langle w\left[\frac{\hat{L}\rho }{\rho }\right]\rangle }_{I},$$

which follows simply from the definition of adjoint operator [11],

(12) $$\int_{V}dx\;w\left(x;t\right)\left(\hat{L}\rho \right)=\int_{V}dx{\left({\hat{L}}^{†}\;w\right)}^{*}\rho \left(x;t\right).$$

Analogously, the CVT allows expectations involving derivatives of the probability density to be rewritten in terms of adjoint operators acting directly on the observables. Thus, after reinterpreting each side as expectations over ρ of suitable observables, by equating the left-hand sides of Equations (10) and (11), we finally arrive at the central identity underlying the framework in Equation (6).

In the case of a continuity equation, Equation (6) reduces to

(13) $$\partial_{t}{\langle w\rangle }_{t}={\langle \partial_{t}w+v\cdot \nabla w\rangle }_{t}={\langle D_{t}w\rangle }_{t},$$

which is the statement that the partial derivative of an expectation is the expectation of the material (convective) derivative.

## 3. Examples

As illustrative examples, we apply the Ehrenfest-type expectation framework developed in the previous section to different evolution operators $\hat{L}$. The purpose of these examples is mainly to show how observable evolution equations can be systematically derived within the formalism, rather than to provide complete predictive models for specific systems. To apply this procedure easily, we use two theorems [8].

(14) $${\langle w\partial_{x}\ln\rho \rangle }_{t}=-{\langle \partial_{x}w\rangle }_{t}$$

(15) $${\langle w\partial_{t}\ln\rho \rangle }_{t}=\partial_{t}{\langle w\rangle }_{t}-{\langle \partial_{t}w\rangle }_{t}.$$

We also use the following relations, which allow us to generate the structures where we can apply the aforementioned identities.

(16) $$\begin{array}{c} \begin{array}{cc} \frac{\partial_{t}\rho }{\rho }= & \partial_{t}\ln\rho \\ \frac{\partial_{i}\rho }{\rho }= & \partial_{i}\ln\rho \\ \frac{\partial_{i}\partial_{j}\rho }{\rho }=\partial_{i}\partial_{j}\ln\rho \;+ & \partial_{j}\ln\rho \;\partial_{i}\ln\rho \;. \end{array} \end{array}$$

This set of Equations (14)–(16) allows us to rewrite the evolution equations in terms of expectation values of observables and their derivatives.

### 3.1. Fokker-Planck Equation

The Fokker-Planck (FP) equations are fundamental in describing the temporal evolution of probability density functions (PDFs) within complex stochastic systems, with applications across diverse domains, including physical and biological modeling [12]. Over the past few decades, numerically solving these equations has constituted a significant area of research. There are different approaches to solving this equation, but each presents its problems and difficulties [13,14]. Motivated by these considerations, we illustrate how the present expectation-value framework can be applied to derive evolution equations for observables governed by Fokker-Planck dynamics.

The n-dimensional Fokker-Planck equation can be written in terms of the operators,

(17) $$-\partial_{t}\rho -[\partial_{i}\mu^{i}-\partial_{j}\partial_{i}D^{ij}]\rho =0.$$

where $\mu^{i}$ and $D^{ij}$ are first-order and second-order tensors, respectively. We emphasize that this form is mathematically equivalent to the standard representation of the Fokker-Planck equation, $\partial_{t}\rho =L\rho$, with L the associated evolution operator. Here, we have simply rearranged all terms to one side of the equation to highlight the structure of the differential operator. This change of sign is purely notational and does not affect the underlying dynamics or interpretation of the equation. The Equation (17), written as a PDE, is given by

$$-\partial_{t}\rho -\rho \partial_{i}\mu^{i}-\mu^{i}\partial_{i}\rho +\rho \partial_{j}\partial_{i}D^{ij}+\partial_{j}\rho \partial_{i}D^{ij}+D^{ij}\partial_{j}\partial_{i}\rho +\partial_{i}\rho \partial_{j}D^{ij}=0\;.$$

Then, operating with ∫dxw and using the set of relations in Equation (16) we obtain:

$$\begin{array}{cc} \langle -w\partial_{t}\ln\rho & -w\partial_{i}\mu^{i}-w\mu^{i}\partial_{i}\ln\rho +w\partial_{j}\partial_{i}D^{ij}+w\partial_{i}D^{ij}\partial_{j}\ln\rho \\ & +wD^{ij}\partial_{j}\partial_{i}\ln\rho +wD^{ij}\partial_{j}\ln\rho \partial_{i}\ln\rho +w\partial_{j}D^{ij}\partial_{i}\ln\rho \rangle =0\; \end{array}$$

Finally, using the CVT and FDT (Equations (14) and (15), respectively) repeatedly, we change the expectation of lnρ, and canceling the identical terms we obtain

(18) $$\partial_{t}\langle w\rangle =\langle \partial_{t}w+\mu^{i}\partial_{i}w+D^{ij}\partial_{j}\partial_{i}w\rangle \;.$$

This relation provides the evolution equation for the expectation value of an arbitrary observable under Fokker-Planck dynamics (for a more detailed derivation, see Appendix B). Note that this equation is not closed in general, as the right-hand side may involve expectations of nonlinear or higher-order functions of the state variables. Closure requires further assumptions or approximations.

It could be interesting to study the time evolution for a fluctuation of a quantity η, so we will consider the fluctuation as follows,

(19) $$\Delta \eta^{2}=\langle \eta^{2}\rangle -{\langle \eta \rangle }^{2}\;.$$

When considering fluctuations of a quantity being measured in a system, we are typically interested in understanding how that quantity varies over time or under different conditions. Fluctuations provide insights into the dynamic behavior and stability of the system, response to external forces, transport properties, and more. By studying these fluctuations, we can gain a deeper understanding of both equilibrium and non-equilibrium processes in systems.

Taken the temporal derivative over Equation (19). Namely,

(20) $$\partial_{t}\;\Delta \eta^{2}=\partial_{t}\langle \eta^{2}\rangle -2\langle \eta \rangle \partial_{t}\langle \eta \rangle ,$$

using Equation (18) and choosing as observable w=η and $w=\eta^{2}$, we can build the terms $\partial_{t}\langle \eta \rangle$ and $\partial_{t}\langle \eta^{2}\rangle$ respectively,

(21) $$\partial_{t}\langle \eta \rangle =\langle \partial_{t}\eta +\mu^{i}\partial_{i}\eta +D^{ij}\partial_{j}\partial_{i}\eta \rangle ,$$

(22) $$\partial_{t}\langle \eta^{2}\rangle =2\langle \eta \partial_{t}\eta +\eta \mu^{i}\partial_{i}\eta +\eta D^{ij}\partial_{j}\partial_{i}\eta +D^{ij}\partial_{j}\eta \partial_{i}\eta \rangle .$$

Then, replacing Equations (21) and (22) in Equation (20), we obtain:

(23) $$\begin{array}{cc} \frac{\partial_{t}\;\Delta \eta^{2}}{2}=\langle D^{ij}\partial_{j}\eta \partial_{i}\eta \rangle & +\langle \eta \left(\partial_{t}\eta +\mu^{i}\partial_{i}\eta +D^{ij}\partial_{j}\partial_{i}\eta \right)\rangle \\ \; & -\langle \eta \rangle \langle \partial_{t}\eta +\mu^{i}\partial_{i}\eta +D^{ij}\partial_{j}\partial_{i}\eta \rangle .\; \end{array}$$

Finally, using the covariance definition,

(24) $$\mathrm{cov}\left(X,Y\right)=\langle XY\rangle -\langle X\rangle \langle Y\rangle ,$$

we rewrite Equation (23) as

$$\frac{\partial_{t}\;\Delta \eta^{2}}{2}=\langle D^{ij}\partial_{j}\eta \partial_{i}\eta \rangle +\mathrm{cov}\left(\eta ,\partial_{t}\eta +\mu^{i}\partial_{i}\eta +D^{ij}\partial_{j}\partial_{i}\eta \right)\;.$$

This result provides a formal evolution equation for the fluctuations of an arbitrary observable in a non-equilibrium system governed by the Fokker-Planck equation. As in the expectation-value case, the resulting equation is not generally closed, since it may involve higher-order correlations or derivatives depending on the observable considered. Traditional analyses often derive moment equations by multiplying the Fokker–Planck equation by powers of the state variables and integrating over the entire phase space. Although effective, this procedure is limited to polynomial observables and requires separate derivations for each moment, often relying on a truncated hierarchy.

In contrast, the method presented here allows us to directly obtain the evolution equation for the observable of interest. This can correspond to a statistical moment, a nonlinear function, or a physically relevant quantity, and does not require constructing the hierarchy moment by moment or explicitly solving the full probability-density equation. Furthermore, since the approach is derived from the general linear evolution equation introduced in Equation (4), it applies to a broader class of systems beyond the Fokker–Planck case. Its generality and directness make it particularly useful in situations where the full probability distribution is not available or where non-standard observables are of interest.

To illustrate the practical application of these general equations, we present examples that focus on estimating the diffusion coefficient. For this purpose, we have selected a relevant physical system: the Earth’s outer radiation belt, where the Earth’s dipolar magnetic field confines charged particles. During typical geomagnetic phenomena, such as geomagnetic storms, variations in the magnetic field alter the third adiabatic invariant, leading to radial diffusion in dipolar coordinates, where the *L*-shell represents the radial position in dipolar coordinates. This phenomenon is particularly important because the diffusion coefficient $D^{LL}$ in the dipolar radial direction is challenging to estimate using conventional methods.

If we take the Equation (18), and if we choose as an arbitrary observable $w=\Delta L^{2}$ or even take the Equation (23) and choose as an observable η=L, several simplifications can be made. First, since *L* does not explicitly depend on time, we set $\partial_{t}L=0$. In addition, for purely radial diffusion, we neglect the drift term $\mu^{i}\partial_{i}L$, while the second derivative $\partial_{i}\partial_{j}L$ vanishes because *L* is a linear function of the dipolar coordinates. Finally, we assume that $D^{LL}$ can be factored outside the expectation value, as it is either homogeneous or weakly varying within the narrow region of phase space considered. Under these assumptions, we recover the classical result for the diffusion coefficient in the radial dipole direction given by [15] $D^{LL}=\frac{\langle \Delta L^{2}\rangle }{\tau }$ which corresponds to the result of the diffusion coefficient associated with the mean squared displacement in a Brownian motion.

It is worth emphasizing that, within the present framework, no specific parametric form of the probability distribution is assumed in deriving the observable evolution equations. In the example considered here, the diffusion coefficient in the dipolar radial direction $D^{LL}$ emerges from the evolution of the second moment $\langle \Delta L^{2}\rangle$ under the simplifying assumptions discussed above. In this sense, the framework recovers the standard diffusion result without requiring the explicit analytical solution or reconstruction of the full probability-density equation.

The purpose of this example is mainly illustrative and intended as a consistency check showing that the Ehrenfest-type formalism reproduces known diffusion relations under commonly used approximations. In more complex situations, where the underlying probability distribution may be unknown or difficult to reconstruct explicitly, the observable-based formulation may still provide a useful reduced description in terms of directly measurable quantities such as $\langle \Delta L^{2}\rangle$.

In practical terms, the framework suggests a possible route for estimating effective diffusion coefficients from observational data through the temporal evolution of suitable observables. For instance, satellite missions monitoring the Earth’s radiation belts routinely provide time series associated with the radial position *L* of charged particles. Under the approximations discussed above, one may estimate the evolution of $\langle \Delta L^{2}\rangle$ directly from such observations and relate it to an effective diffusion coefficient $D^{LL}$ without explicitly solving the full Fokker-Planck equation.

We emphasize, however, that the applicability of this procedure depends on the validity of the approximations involved and should therefore be interpreted as an illustrative example of the observable-based framework rather than as a complete transport model.

### 3.2. Liouville Equation

The Liouville equation stands as a cornerstone in theoretical physics, providing a rigorous description of the temporal evolution of physical systems in phase space [16]. Originating in classical statistical mechanics and extended to quantum mechanics, this equation plays a central role in predicting the behavior of complex systems and finds utility across diverse contexts [17,18]. By delineating how particle position and momentum distributions evolve over time, the Liouville equation not only furnishes profound insight into the dynamics of physical and chemical systems but also underpins the formulation of fundamental conservation principles in physics [19].

The Liouville conservation theorem in phase space can be written as:

$$\partial_{t}\rho +\left\{\rho ,H\right\}=0$$

H:H(q,p)

ρ:ρ(q,p,t),

where *H* is Hamiltonian in the phase space, ρ is probability density in the same space and {·,·} is the Poisson bracket. Expanding the Poisson bracket, we obtain a PDE for the Liouville equation,

$$\partial_{t}\rho +\partial_{q}\rho \partial_{p}H-\partial_{p}\rho \partial_{q}H=0\;.$$

Applying the same expectation-value framework introduced in the previous section, we build an equivalent PDE for lnρ using the set shown in Equation (16), to obtain

(25) $$\langle w\partial_{t}\ln\rho +w\partial_{p}H\;\partial_{q}\ln\rho -w\partial_{q}H\partial_{p}\ln\rho \rangle =0\;.$$

Again, using the CVT and FDT shown in Equations (14) and (15), and removing opposite terms from the Equation (25), we obtain the corresponding evolution equation for the expectation value of an arbitrary observable under Liouville dynamics,

$$\partial_{t}\langle w\rangle =\langle \partial_{t}w+\partial_{q}w\;\partial_{p}H-\partial_{p}w\;\partial_{q}H\rangle \;.$$

This equation, rewritten in Poisson brackets terms is as follows

(26) $$\frac{\partial }{\partial t}\langle w\rangle =\langle \frac{\partial w}{\partial t}+\left\{w,H\right\}\rangle \;,$$

which constitutes the classical counterpart of the Ehrenfest relation in quantum mechanics [20],

(27) $$\frac{d}{dt}\langle \hat{\Omega }\rangle =\langle \frac{\partial \hat{\Omega }}{\partial t}-\frac{i}{\hbar }\left[\hat{\Omega },\hat{H}\right]\rangle ,$$

where the quantum-mechanical commutator has replaced the Poisson bracket.

Now if we compute the time evolution of a quantity in a system that follows the Liouville equation, build as in the FPE case, $\partial_{t}\langle \eta \rangle$ and $\partial_{t}\langle \eta^{2}\rangle$, from Equation (26),

(28) $$\frac{\partial_{t}\;\Delta \eta^{2}}{2}=\langle \eta \left(\partial_{t}\eta +\left\{\eta ,H\right\}\right)\rangle -\langle \eta \rangle \langle \partial_{t}\eta +\left\{\eta ,H\right\}\rangle ,$$

considering the definition of total temporal derivative for a quantity,

$$\frac{d\eta }{dt}=\dot{\eta }=\partial_{t}\eta +\left\{\eta ,H\right\},$$

we use Equation (24) to rewrite Equation (28) as follow,

$$\frac{\partial_{t}\;\Delta \eta^{2}}{2}=\langle \eta \;\dot{\eta }\rangle -\langle \eta \rangle \langle \dot{\eta }\rangle ,$$

(29) $$\frac{\partial_{t}\;\Delta \eta^{2}}{2}=\mathrm{cov}\left(\eta ,\dot{\eta }\right)\;,$$

which provides the evolution equation for the fluctuations of an arbitrary observable under Liouville dynamics. As in the Fokker-Planck case, the resulting equations are not generally closed and may involve higher-order correlations depending on the observable considered. The purpose of the present derivation is therefore not to provide a universal closure prescription, but rather to illustrate how the Ehrenfest-type framework can systematically generate observable evolution equations directly from the underlying Liouville dynamics.

## 4. Discussion

In this work, we formulated a systematic expectation-value framework for dynamical systems whose probability densities evolve according to linear partial differential equations, including Liouville-, Fokker–Planck-, and continuity-type dynamics. The formalism provides evolution equations for arbitrary differentiable observables and fluctuations directly from the underlying probability-density dynamics, establishing a classical Ehrenfest-type relation for observable evolution in nonequilibrium statistical mechanics.

The framework is based on expectation-calculus identities associated with the Conjugate Variables Theorem and the Fluctuation–Dissipation Theorem, which allow derivatives of the probability density to be systematically rewritten in terms of expectation values of observables and adjoint operators. In contrast to traditional moment methods, which are typically formulated in terms of polynomial observables and require the hierarchy to be constructed moment by moment, the present formulation is expressed directly in terms of arbitrary observables, including nonlinear observables and fluctuation-related quantities. In this sense, the formalism provides a unified operational framework for deriving observable evolution equations without explicitly solving the full probability-density equation.

At the same time, the resulting equations are not closed in general, since they typically involve higher-order moments, correlations, or derivatives depending on the observable considered. This can be seen, for instance, in Equation (23), where the evolution of the variance involves terms such as $\langle D^{ij}\partial_{j}\eta ,\partial_{i}\eta \rangle$ and $\langle \eta ,\partial_{j}\partial_{i}\eta \rangle$, which depend on higher-order correlations beyond the second moment.

Accordingly, the present work is not intended as a universal closure scheme or as a complete reduced description for arbitrary nonequilibrium systems. Rather, it should be understood as a systematic observable-based formulation from which problem-dependent approximations, closure assumptions, or reduced dynamical descriptions may be constructed. In many practical situations, the relevant higher-order quantities may be directly estimated from simulations, experiments, or observational data, or approximated using established modeling techniques. In this sense, the framework may provide a useful operational setting for constructing reduced descriptions in systems where the full probability-density dynamics are inaccessible.

The examples presented for Fokker-Planck and Liouville dynamics mainly serve to illustrate how the formalism systematically generates evolution equations for observables under different dynamical operators. In particular, the radiation-belt example demonstrates that the framework is consistent with standard diffusion relations under commonly used approximations, while avoiding the explicit reconstruction of the full probability-density evolution. In this context, observable-based quantities such as $\langle \Delta L^{2}\rangle$ may be directly estimated from observational data, suggesting possible applications of the formalism to reduced descriptions of transport processes in complex systems.

Related applications of the present observable-based formalism have previously been explored in the context of Vlasov dynamics and adiabatic invariants in magnetized plasmas [21,22], where the resulting evolution equations were analyzed numerically for specific observables. These studies illustrate that, although the closure problem remains in general, suitable observables, approximations, or externally accessible quantities extracted from simulations, experiments, or observational data may provide practical ways to construct reduced descriptions within the framework. In this sense, the formalism may be particularly useful in situations where the full probability-density dynamics are inaccessible, but relevant observables can still be measured or estimated reliably.

Future work should focus on developing systematic approximation and closure strategies within the present observable-based framework, as well as exploring applications to systems where only partial information about the underlying dynamics is experimentally accessible. Potential directions include fluctuation dynamics in kinetic plasmas, reduced descriptions of transport processes, and data-driven estimation of effective evolution equations for selected observables. We believe that, within these limitations, the Ehrenfest-type framework presented here provides a useful and conceptually transparent step toward systematic observable dynamics in nonequilibrium statistical mechanics.

## Data Availability

No new data were created or analyzed in this study. Data sharing is not applicable to this article.

## Abbreviations

The following abbreviations are used in this manuscript:

NESM	Non-Equilibrium Statistical Mechanics
PDEs	Partial Differential Equations
FPE	Fokker-Planck Equation
CVT	Conjugate Variables Theorem
FDT	Fluctuation-Dissipation Theorem

## Appendix A. Fluctuation-Dissipation Theorem

Let us now take the derivative of the expectation ${\langle \omega \left(x,\lambda \right)\rangle }_{\lambda }$ with respect to one of the parameters, $\lambda_{j}$:

$$\frac{\partial }{\partial \lambda_{j}}{\langle \omega \rangle }_{\lambda }=\frac{\partial }{\partial \lambda_{j}}\left(\int dx\;\omega (x,\lambda )P(x|\lambda )\right)$$

$$=\int dx\left[P\left(x|\lambda \right)\frac{\partial \omega }{\partial \lambda_{j}}+\omega \frac{\partial }{\partial \lambda_{j}}P\left(x|\lambda \right)\right].$$

Rewriting the second term as an expectation, we obtain:

(A1) $$\frac{\partial }{\partial \lambda_{j}}{\langle \omega \rangle }_{\lambda }={\langle \frac{\partial \omega }{\partial \lambda_{j}}\rangle }_{\lambda }+{\langle \omega \frac{\partial }{\partial \lambda_{j}}\ln P\left(x|\lambda \right)\rangle }_{\lambda },$$

which resembles the CVT, as it involves a “free” function ω(x,λ). This Equation (A1) is the most general form of a family of “fluctuation theorems” [8]. The special case where the probability distribution P(x|λ) is a Maximum Entropy model was previously derived by Jaynes in his book [10], and is given by:

$$P\left(x|\lambda \right)=\frac{1}{Z(\lambda )}P_{0}\left(x\right)\exp\left(-\sum_{j=1}^{m}\lambda_{j}f_{j}\left(x\right)\right),$$

where Z(λ) is the partition function, $P_{0}\left(x\right)$ is the prior distribution, and $\lambda_{j}$ are the parameters associated with the functions $f_{j}\left(x\right)$. Then,

(A2) $$\frac{\partial }{\partial \lambda_{j}}\ln P\left(x|\lambda \right)=-f_{j}+{\langle f_{j}\rangle }_{\lambda }\;,$$

since,

$$\frac{\partial }{\partial \lambda_{j}}\ln Z\left(\lambda \right)={\langle f_{j}\rangle }_{\lambda }\;.$$

Substituting Equation (A2) into Equation (A1),

$$\frac{\partial }{\partial \lambda_{j}}{\langle \omega \rangle }_{\lambda }={\langle \frac{\partial \omega }{\partial \lambda_{j}}\rangle }_{\lambda }+\langle \omega \left(-f_{j}+{\langle f_{j}\rangle }_{\lambda }\right)\rangle ,$$

Assuming ω does not explicitly depend on λ,

$$\frac{\partial }{\partial \lambda_{j}}{\langle \omega \rangle }_{\lambda }=-\left({\langle \omega f_{j}\rangle }_{\lambda }-{\langle \omega \rangle }_{\lambda }{\langle f_{j}\rangle }_{\lambda }\right),$$

finally we can rewrite the previous equation that follows,

(A3) $$\frac{\partial }{\partial \lambda_{j}}{\langle \omega \rangle }_{\lambda }=-{\langle \delta \omega \delta f_{j}\rangle }_{\lambda }\;,$$

where we define the fluctuations,

$$\delta \omega =\omega -{\langle \omega \rangle }_{\lambda },$$

$$\delta f_{j}=f_{j}-{\langle f_{j}\rangle }_{\lambda },$$

Here, the right-hand side of Equation (A3) represents a covariance between ω and $f_{j}$, showing that the magnitude of its fluctuations governs the response of an observable to variations in $\lambda_{j}$. This recovers the classical form of the fluctuation–dissipation theorem [23], which connects the linear response of a system to external perturbations with the internal equilibrium fluctuations.

For instance, in the canonical ensemble, where λ=β and the observable conjugate to this parameter is the energy H(x), our general relation (A3) becomes,

$$\frac{\partial \langle H\rangle }{\partial \beta }=-\langle \delta H^{2}\rangle =-\mathrm{Var}\left(H\right)\;,$$

which is a well-known fluctuation identity that relates energy fluctuations to the heat capacity [24,25]. This demonstrates that our result not only generalizes fluctuation relations for arbitrary observables and parameters, but also includes as special cases the standard expressions encountered in statistical mechanics.

## Appendix B. Derivation of the Observable Evolution Equation for the Fokker–Planck Case

In this Appendix, we provide the explicit derivation of the evolution equation for the expectation value of a general w(x;t), in the case where a Fokker–Planck equation governs the underlying dynamics. This computation illustrates how our formalism applies in this context and clarifies the analytical structure of the result discussed in the main text.

We begin by expanding the Fokker–Planck operator acting on the probability density,

$$-\partial_{t}\rho -\rho \partial_{i}\mu^{i}-\mu^{i}\partial_{i}\rho +\rho \partial_{j}\partial_{i}D^{ij}+\partial_{j}\rho \partial_{i}D^{ij}+D^{ij}\partial_{j}\partial_{i}\rho +\partial_{i}\rho \partial_{j}D^{ij}=0\;.$$

Then, operating with ∫dxw and using the set of relations in Equation (16) we obtain:

$$\begin{array}{cc} \langle -w\partial_{t}\ln\rho & -w\partial_{i}\mu^{i}-w\mu^{i}\partial_{i}\ln\rho +w\partial_{j}\partial_{i}D^{ij}+w\partial_{i}D^{ij}\partial_{j}\ln\rho \\ & +wD^{ij}\partial_{j}\partial_{i}\ln\rho +wD^{ij}\partial_{j}\ln\rho \partial_{i}\ln\rho +w\partial_{j}D^{ij}\partial_{i}\ln\rho \rangle =0\;. \end{array}$$

In the following, we isolate and analyze each term in the equation above to construct the evolution identity. To carry out this derivation, we apply the general expectation identities presented in Equations (14) and (15), which correspond to the Conjugate Variable Theorem (CVT) and the fluctuation–dissipation theorems (FDT), whenever applicable.

$$\begin{array}{cc} 1^{\circ }-\langle w\;\partial_{t}\ln\rho \rangle & =-\partial_{t}\langle w\rangle +\langle \partial_{t}w\rangle \\ 2^{\circ }\langle -w\;\partial_{i}\mu^{i}\rangle & =\langle -w\;\partial_{i}\mu^{i}\rangle \\ 3^{\circ }\langle -w\;\mu^{i}\;\partial_{i}\ln\rho \rangle & =-\langle -\partial_{i}\left(w\mu^{i}\right)\rangle \\ \; & =\langle \mu^{i}\;\partial_{i}w+w\;\partial_{i}\mu^{i}\rangle \\ 4^{\circ }\langle w\;\partial_{j}\partial_{i}D^{ij}\rangle & =\langle w\;\partial_{j}\partial_{i}D^{ij}\rangle \\ 5^{\circ }\langle w\;\partial_{i}D^{ij}\;\partial_{j}\ln\rho \rangle & =-\langle \partial_{j}\left(w\;\partial_{i}D^{ij}\right)\rangle \\ \; & =-\langle \partial_{j}w\;\partial_{i}D^{ij}+w\;\partial_{j}\partial_{i}D^{ij}\rangle \end{array}$$

$$6^{\circ }\langle wD^{ij}\partial_{j}\partial_{i}\ln\rho \rangle$$

$$\begin{array}{cc} 7^{\circ }\langle wD^{ij}\partial_{j}\ln\rho \;\partial_{i}\ln\rho \rangle \; & =-\langle \partial_{i}\left(wD^{ij}\partial_{j}\ln\rho \right)\rangle \\ \; & =-\langle \partial_{i}w\;D^{ij}\partial_{j}\ln\rho +w\;\partial_{i}D^{ij}\;\partial_{j}\ln\rho +wD^{ij}\partial_{i}\partial_{j}\ln\rho \rangle \\ \; & =-\langle -\partial_{j}\left(\partial_{i}w\;D^{ij}\right)-\partial_{j}\left(w\;\partial_{i}D^{ij}\right)+wD^{ij}\partial_{i}\partial_{j}\ln\rho \rangle \\ \; & =\begin{array}{cc} -\langle -\left(D^{ij}\partial_{j}\partial_{i}w+\partial_{j}D^{ij}\partial_{i}w\right)-(\partial_{j}w\;\partial_{i}D^{ij} & +w\;\partial_{j}\partial_{i}D^{ij})\\ \; & +wD^{ij}\partial_{i}\partial_{j}\ln\rho \rangle \end{array}\\ \; & =\langle D^{ij}\partial_{j}\partial_{i}w+\partial_{j}D^{ij}\partial_{i}w+\partial_{j}w\;\partial_{i}D^{ij}+w\;\partial_{j}\partial_{i}D^{ij}-wD^{ij}\partial_{i}\partial_{j}\ln\rho \rangle \end{array}$$

$$\begin{array}{cc} 8^{\circ }\langle w\partial_{j}D^{ij}\;\partial_{i}\ln\rho \rangle \; & =-\langle \partial_{i}\left(w\partial_{j}D^{ij}\right)\rangle \\ \; & =-\langle \partial_{i}w\;\partial_{j}D^{ij}+w\;\partial_{i}\partial_{j}D^{ij}\rangle . \end{array}$$

After applying the corresponding expectation theorems to each term, we can rewrite the full equation in terms of the new structures obtained. At this stage, we proceed to reorganize the expression and cancel identical contributions with opposite signs. In doing so, we have explicitly assumed the commutativity of mixed derivatives, i.e., $\partial_{i}\partial_{j}=\partial_{j}\partial_{i}$, which holds under the smoothness conditions of the functions involved. Collecting the surviving contributions, the equation reduces to:

−∂t〈w〉+〈∂tw−w∂iμia+μi∂iw+w∂iμia+w∂j∂iDijb−∂jw∂iDijc+wDij∂i∂jlnρd−bw∂j∂iDijb+Dij∂j∂iw+∂jDij∂iwe+∂jw∂iDijc+w∂i∂jDijf−wDij∂i∂jlnρd−∂jDij∂iwe−w∂i∂jDijf=0,

(A4) $$\partial_{t}\langle w\rangle =\langle \partial_{t}w+\mu^{i}\partial_{i}w+D^{ij}\partial_{j}\partial_{i}w\rangle \;.$$
